# Diagnosing SARS-CoV-2 with Antigen Testing, Transcription-Mediated Amplification and Real-Time PCR

**DOI:** 10.3390/jcm10112404

**Published:** 2021-05-29

**Authors:** Sascha Dierks, Oliver Bader, Julian Schwanbeck, Uwe Groß, Michael S. Weig, Kemal Mese, Raimond Lugert, Wolfgang Bohne, Andreas Hahn, Nicolas Feltgen, Setare Torkieh, Fenja R. Denker, Peer Lauermann, Marcus W. Storch, Hagen Frickmann, Andreas Erich Zautner

**Affiliations:** 1 Institute for Clinical Chemistry, University Medical Center Göttingen, 37075 Göttingen, Germany; sascha.dierks@med.uni-goettingen.de; 2 Institute for Medical Microbiology, University Medical Center Göttingen, 37075 Göttingen, Germany; obader@gwdg.de (O.B.); julian.schwanbeck@med.uni-goettingen.de (J.S.); ugross@gwdg.de (U.G.); mweig@gwdg.de (M.S.W.); kemal.mese@med.uni-goettingen.de (K.M.); rlugert@gwdg.de (R.L.); wbohne@gwdg.de (W.B.); 3 Institute for Medical Microbiology, Virology and Hygiene, University Medicine Rostock, 18057 Rostock, Germany; andreas.hahn@uni-rostock.de (A.H.); frickmann@bnitm.de (H.F.); 4 Department of Ophthalmology, University Medical Center Göttingen, 37075 Göttingen, Germany; nicolas.feltgen@med.uni-goettingen.de (N.F.); setare.torkieh@stud.uni-goettingen.de (S.T.); fenjaruth.denker@stud.uni-goettingen.de (F.R.D.); peer.lauermann@med.uni-goettingen.de (P.L.); marcus.storch@med.uni-goettingen.de (M.W.S.); 5 Department of Microbiology and Hospital Hygiene, Bundeswehr Hospital Hamburg, 20359 Hamburg, Germany

**Keywords:** SARS-CoV-2, rapid diagnostic resting, antigen test comparison, transcription-mediated amplification TMA, real-time PCR

## Abstract

This study was performed as a head-to-head comparison of the performance characteristics of (1) two SARS-CoV-2-specific rapid antigen assays with real-time PCR as gold standard as well as (2) a fully automated high-throughput transcription-mediated amplification (TMA) assay and real-time PCR in a latent class analysis-based test comparison without a gold standard with several hundred samples in a low prevalence “real world” setting. Recorded sensitivity and specificity of the NADAL and the LumiraDx antigen assays and the Hologic Aptima SARS-CoV-2 TMA assay were 0.1429 (0.0194, 0.5835), 0.7644 (0.7016, 0.8174), and 0.7157 (0, 1) as well as 0.4545 (0.2022, 0.7326), 0.9954 (0.9817, 0.9988), and 0.9997 (not estimable), respectively. Agreement kappa between the positive results of the two antigen-based assays was 0.060 (0.002, 0.167) and 0.659 (0.492, 0.825) for TMA and real-time PCR. Samples with low viral load as indicated by cycle threshold (Ct) values > 30 were generally missed by both antigen assays, while 1:10 pooling suggested higher sensitivity of TMA compared to real-time PCR. In conclusion, both sensitivity and specificity speak in favor of the use of the LumiraDx rather than the NADAL antigen assay, while TMA results are comparably as accurate as PCR, when applied in a low prevalence setting.

## 1. Introduction

The Severe Acute Respiratory Syndrome Corona Virus 2 (SARS-CoV-2), causing the Corona Virus Disease 2019 (COVID-19) pandemic, was first reported in Wuhan, China, in 2019 [1]. Due to the subsequent rapid global spread, the availability of both rapid and reliable diagnostic approaches became an issue of concern. To match this need, the first in-house real-time PCR protocols were soon provided [2].

However, as traditional real-time PCR requires sophisticated laboratory infrastructure and skilled technical laboratory assistance, there was considerable demand for cartridge-based or otherwise fully-automated, point-of-care-testing (POCT) like devices for the molecular detection of SARS-CoV-2 RNA. Cepheid’s (Sunnyvale, CA, USA) GeneXpert system and Abbott’s (Chicago, IL, USA) ID Now system were the first broadly available assays showing good performance characteristics in a Cochrane analysis published as early as in August 2020 [3,4,5,6,7,8,9,10,11,12,13]. Additionally, Luminex’s (Austin, TX, USA) cartridge-based ARIES PCR system allowed fully automated testing comparably early [14,15]. Another fully automated CE certified and FDA cleared approach for the detection of SARS-CoV-2 nucleic acids from upper respiratory tract specimens is the Hologic Aptima SARS-CoV-2 TMA assay on the Hologic Panther platform [16,17,18,19,20,21,22,23,24]. The “Panther” assay is based on transcription-mediated amplification (TMA) of two targets and provides qualitative results only, without the possibility to gain further information on the virus load of positive samples, unlike with standard real-time PCR-based SARS-CoV2 assays based on Ct (cycle threshold) value assessment.

Although easy to apply with little training effort for laboratory personnel, such fully-automated POCT-like molecular approaches still have considerable limitations. Firstly, many of them [3,4,5,6,7,8,9,10,11,12,13,14,15] are poorly suited for large scale assessments. Secondly, there has been a shortage of consumables from the beginning of the SARS-CoV-2 pandemic. Thirdly, traditional immunochromatographic antigen-based rapid tests were less expensive, and finally, highly sensitive molecular assays tend to detect prolonged residual shedding of SARS-CoV-2 RNA with questionable relevance in terms of transmission risk [25,26,27,28]. Although, accordingly, development efforts were soon directed towards traditional antigen-based rapid tests, performance characteristics of first respective approaches left a lot of room for improvement. In the above-mentioned Cochrane analysis from August 2020 [3], sensitivity of available SARS-CoV-2 antigen tests ranged from 0% to 94% with average sensitivity of 56.2% (95% confidence interval (0.95 CI) 29.5% to 79.8%) and average specificity of 99.5% (0.95 CI 98.1% to 99.9%). Despite the comparably good specificity, according to Bayes’ theorem [29], application in low prevalence settings can lead to both problems regarding the positive as well as the negative predictive value [30], thus limiting the practical application of such early designs for public health interventions.

To identify optimized strategies of antigen-based rapid testing for SARS-CoV-2, numerous studies [31,32,33,34,35,36,37,38,39,40,41,42,43,44,45,46] have been performed and partly summarized in additional reviews [47,48,49,50,51,52,53,54,55,56] after those first, quite disillusioning trials [3]. Despite persisting sensitivity issues and resulting in quite variable diagnostic reliability [57], antigen-testing was broadly used in the reaction to the second SARS-CoV-2 wave in many countries [58]. In case of implementation outside laboratory infrastructure under tropical conditions, environmental conditions such as room temperature and air moisture have also been described to potentially interfere with test reliability [59].

For gradual improvement of the diagnostic quality of rapid SARS-CoV-2 tests, head-to-head comparisons have been suggested in order to optimize diagnostic solutions [36]. In times of increasing spread of virus mutants, it is of further interest to assess effects of mutations on the reliability of antigen testing similar to the S gene target failure, as observed in real-time PCR for the B1.1.7 lineage [60].

In the study conducted here, we sought to individually evaluate the NADAL COVID-19 Ag Test (Nal von Minden GmbH, Regensburg, Germany) and a microfluidic immunofluorescence assay (SARS-CoV-2 Ag Test, LumiraDx GmbH, Cologne, Germany) as well as a head-to-head assessment against real-time PCR. Individual [38,61] and combined [62] assessments of these assays had yielded promising yet still imperfect results in previous preliminary studies [62]. However, the quoted study [62] had been performed with a small sample number of just 74 PCR-positive and 26 PCR-negative specimens yet the resulting preliminary results suggested specificity close to 100% for both rapid assays. While sensitivity of the LumiraDx assay was 50.0% in total and 100% in samples with RNA copy numbers > 10^6^ copies/mL, the values were 24.3% and 76.2% for the NADAL assay, respectively [62].

Further, the fully automated “Panther” TMA assay, for which high sensitivity had been reported in previous studies [16,17,18,19,20,21,22,23,24], was also included in the assessment. While real-time PCR was considered as a reference standard or “gold standard” for the comparison with the antigen assays [29], indirect accuracy estimation by latent class analysis (LCA) [29,63] was chosen for the evaluation of the TMA assay, for which similar or higher sensitivity in comparison to real-time PCR could be expected [16,17,18,19,20,21,22,23,24]. A larger number of several hundred non-preselected samples were analyzed in a low-prevalence setting for both the gold standard-based assessment of the antigen tests and the LCA-based assessment of the TMA platform, to assess the performance of the assays in a diagnostic “real world” scenario.

## 2. Materials and Methods

### 2.1. Samples Population, Inclusion and Exclusion Criteria

A total of 444 employees of the University Medical Center Göttingen were assessed by rapid antigen testing and PCR for respiratory carriage of SARS-CoV-2. Samples were included if they had been analyzed in parallel by at least one antigen test and at least one real-time PCR assay as described below. If these minimum requirements were not fulfilled, the sample was excluded from the assessment. Real-time PCR was considered as the diagnostic reference standard or “gold standard” for this assessment [29].

Further, the Hologic Aptima SARS-CoV-2 TMA assay was validated with 322 diagnostic nasopharyngeal swabs from patients of the University Medical Center Göttingen in comparison to the Genesig COVID-19 Real-Time PCR assay as detailed below in a test comparison without a gold standard [29,63]. Both assays were run in parallel in order to avoid a bias due to loss of sensitivity after sample storage. In order to assess the effects of pooling, 1:10 pools of 18 samples with low viral load (Ct values > 30 in Genesig real-time PCR) were comparatively assessed by both the Genesig real-time PCR and TMA assessment.

For both test comparison approaches, cycle threshold (Ct) values of the real-time PCR assays were included in the assessments.

No data on the participating individuals were collected in the course of this surveillance screening for organizational reasons, an admitted violation of the Standards for Reporting of Diagnostic Accuracy Studies (STARD) [64].

### 2.2. Applied Test Assays

The applied rapid antigen-based or automated molecular SARS-CoV-2 tests comprised the NADAL COVID-19 Ag Test (Nal von Minden GmbH, Regensburg, Germany), the microfluidic immunofluorescence assay LumiraDx SARS-CoV-2 Ag Test (LumiraDx GmbH, Cologne, Germany), and the Hologic Aptima SARS-CoV-2 TMA assay (Hologic Deutschland GmbH, Wiesbaden-Nordenstadt, Germany). All these assays were applied according to the manufacturers’ guidelines [16,17,18,19,20,21,22,23,24,38,61,62]. Comparative PCR testing as gold standard for the antigen test assessment was performed either by the Roche Cobas SARS-CoV-2 PCR (Roche, Basel, Switzerland) [13,65], the Genesig Real-Time PCR Coronavirus (COVID-19) assay (Primerdesign Ltd., Chandlers Ford, UK) [66], or the Cepheid Xpert Xpress SARS-CoV-2 PCR (Cepheid, Sunnyvale, CA, USA) [13,14]. The choice of the PCR assay depended on availability in the diagnostic routine. For the assessment of the Hologic Aptima SARS-CoV-2 TMA assay [16,17,18,19,20,21,22,23,24] in a test comparison without a gold standard [29,63], the Genesig Real-Time PCR Coronavirus (COVID-19) assay (Primerdesign Ltd., Chandlers Ford, UK) [66] was applied only as competing assay.

### 2.3. Statistics

In a descriptive approach, sensitivity and specificity of the rapid test assays was calculated applying the PCR results as gold standard for the antigen test and applying latent class analysis (LCA) [29,63] for the comparison of the TMA assay with real-time PCR. Predictive values were provided based on calculated sensitivity, specificity and prevalence. The software Stata/IC 15.1 for macOS 64-bit Intel (College Station, TX, USA) was used. For the assessment of the agreement between antigen as well as TMA testing results and real-time PCR results, Cohen’s kappa was calculated with the categories poor (below 0.00), slight (0.00–0.20), fair (0.21–0.40), moderate (0.41–0.60), substantial (0.61–0.80) and almost perfect (0.81–1.00) as described [67]. Matching of positive and negative results with Ct-values of real-time PCR was descriptively assessed.

### 2.4. Ethical Clearance

The study was ethically approved by the institutional ethics board of the University Medical Center Göttingen (Application number/project identification code 21/05/20 from 21 May 2020), allowing the fully anonymized use of residual sample materials for test comparison purposes.

## 3. Results

### 3.1. Sensitivity and Specificity of the SARS-CoV-2 Antigen Testing Approaches

The LumiraDx SARS-CoV-2 Ag Test was applied with 444 individuals. Of these, 437 tested negative, and 7 tested positive. Sensitivity and specificity with 0.95 confidence intervals and PCR as gold standard were calculated as 0.4545 (0.2022, 0.7326) and 0.9954 (0.9817, 0.9988), respectively (Table 1).

Additionally, the NADAL COVID-19 Ag Test was applied with 215 individuals. Of these, 164 of them tested negative, 8 tested positive, and for 43 individuals there was no clear test result. Sensitivity and specificity with 0.95 confidence intervals were calculated as 0.1429 (0.0194, 0.5835) and 0.7644 (0.7016, 0.8174), respectively, with PCR as gold standard (Table 1).

### 3.2. Agreement According to Cohen’s Kappa between the Antigen Assays and Real-Time PCR

The kappa coefficient with 0.95 confidence interval between the two rapid tests was calculated as 0.060 (0.002, 0.167) (Table 1).

### 3.3. Cycle Threshold (Ct) Values of Real-Time PCR in Samples with Positive and Negative Antigen Test Results

Low numbers of positive samples allowed a superficial assessment of Ct values only. However, as indicated in Table 2, Ct values ≥ 30 were associated with falsely negative results in the assessed antigen tests.

### 3.4. Sensitivity and Specificity of the Hologic Aptima SARS-CoV-2 TMA Assay and the Genesig Real-Time PCR Assay as Calculated by LCA and Agreement According to Cohen’s Kappa between the TMA Assay and Real-Time PCR

When comparing the TMA assay with the Genesig real-time PCR assay using LCA, both assays showed excellent specificity close to 100%. Agreement of positive results as indicated by Cohen’s kappa was substantial [67]. Calculated sensitivity of the TMA assays (71.6%) was slightly better than the sensitivity of the real-time PCR assay (65.4%) with the sample population assessed (Table 3).

### 3.5. Cycle Threshold (Ct) Values of Genesig Real-Time PCR in Samples with Positive and Negative TMA Test Results

The mean value of Ct values of the Genesig real-time PCR for samples concordantly positive in the TMA assay and the real-time PCR assay was 30.8. For samples positive in real-time PCR but negative in the TMA assay, this mean value was 38.8 and thus close to the detection threshold (Table 4).

### 3.6. Sensitivity of TMA and Genesig Real-Time PCR in Case of 1:10 Pooling of Samples with Low Viral Loads and Comparison with Genesig Real-Time PCR Ct Values

In case of 1:10 pooling of 18 samples with low viral loads (Ct > 30 in the Genesig real-time PCR), TMA was considerably more sensitive (83.3%) than Genesig real-time PCR (33.3%) with only slight concordance [67] (Table 5). Samples with positive results in Genesig real-time PCR after pooling showed Ct values smaller than 36 without exemption, resulting in a mean Ct value of 38.2 after pooling (Table 6).

### 3.7. Predictive Value as Calculated Based on Sensitivity, Specificity of the Test as Well as the Prevalence in the Assessed Population

By applying LCA with the data from the test comparison of TMA and Genesig real-time PCR testing, a prevalence of 9.95% was calculated. Based on the sensitivities and specificities as estimated for the different compared test assays, reduced positive but high negative predictive values of the antigen tests were observed, as expected for a low prevalence setting. Additionally, due to the calculated high specificity, the positive predictive values were high for the molecular assays (Table 7).

## 4. Discussion

In this study we assessed the diagnostic performance of the antigen-based rapid test assays NADAL and LumiraDx under “real-world” conditions. As expected from previous assessments [38,62,63], the applied antigen test assays were more likely to identify samples with higher pathogen load, as indicated by Ct values < 30, while samples with higher Ct values were missed. This result is not surprising and confirms the suitability of rapid antigen testing for the identification of individuals with high pathogens loads and, therefore an associated high likeliness of transmission only.

More interesting is the merely slight agreement between the positive antigen testing results when directly comparing the two antigen test assays. Certainly, this result is affected by the low number of positive results in the population assessed, nevertheless, it suggests a high variance regarding the positive results that can be expected in a low prevalence community, depending on the antigen test applied.

The recorded sensitivities of the antigen tests were slightly lower in this “real-world” assessment but nevertheless in a similar range with previous evaluation studies [38,62,63]. Regarding specificity however, only the LumiraDx approach showed similar values compared with previous evaluation studies [62,63]. The NADAL assay, in contrast, scored considerably worse [38,63], with specificity as low as 76.4% in the low prevalence population assessed. Due to Bayes’ theorem [29], this will lead to a considerable proportion of falsely positive test results and thus to a poor positive predictive value when applied in a low prevalence setting. So, based on both sensitivity and specificity results, the use of the LumiraDx assay is preferable compared to the NADAL assay.

Furthermore, comparable diagnostic accuracy of Genesig real-time PCR and Hologic Aptima TMA assay, both targeting ORF-1 sequences of SARS-CoV-2 [16,17,18,19,20,21,22,23,24,66], was demonstrated with clinical samples of patients of the University Medical Center Göttingen. The results are in line with previous studies [16,17,18,19,20,21,22,23,24,66] and confirm the suitability of the Hologic Panther TMA platform for fully automated, high-throughput SARS-CoV-2 screening. The low sensitivity of both assays within the assessed sample population is most likely due to the high proportion of samples with low viral loads close to the diagnostic detection threshold. In case of pooling of samples with low viral loads, moderately better sensitivity of the TMA approach compared to the Genesig real-time PCR assay was demonstrated. Despite this better sensitivity, the limitation of lacking semi-quantification when applying the TMA technology remains, as no Ct values are provided. So, TMA assessment may be a powerful tool for high-throughput initial screening for SARS-CoV-2 but is unsuitable for follow-up assessments.

The study has a number of limitations. Firstly, although several hundred individuals could be included in the assessment, the proportion of positives was quite low, making any calculations regarding sensitivity doubtful. As expected for the antigen tests due to the low prevalence, lower positive than negative predictive values were calculated. For the molecular assays, positive predictive values were good due to their high specificity. Furthermore, the combination of low sample count and low prevalence prevented a more sophisticated comparison of rapid test positivity with semi-quantification, as indicated by Ct values in PCR. Secondly, not all samples could be tested with all assays for logistical reasons, which limits their comparability. Thirdly, ethical clearance allowed fully anonymized use of patient samples for the applied test comparison purposes only, so patient-specific features can neither be shown nor assessed.

## 5. Conclusions

Despite the above-mentioned limitations, the presented “real world” assessment suggests superiority of the LumiraDx assay compared to the NADAL assay when applied in a low prevalence setting, based on both sensitivity and specificity considerations. Infections with low viral load, however, will be missed by both of them. In contrast, acceptable sensitivity and diagnostic accuracy similar to real-time PCR, even in the case of 1:10 pooling, could be shown for the Hologic Aptima SARS-CoV-2 TMA assay for high- throughput screenings.

Other than in previous assessments with pre-selected positive and negative samples [63], our assessment provides a “real-life experience” in a low prevalence setting, suggesting that test accuracy might vary under such conditions compared to the results of standardized study settings. Large confidence intervals, which admittedly limit the interpretability of the data, are due to financial restrictions of investigator-initiated studies such as the one presented here. Of course, generously funded studies have the advantage of larger sample sizes, which in turn will result in lower 0.95 CI-intervals as suggested recently [63].

Cross-reactions due to usually unknown co-infections are always possible in assessments in real-life situations, which describe the diagnostic performance of test assays that can actually be expected in contrast to possible performance under idealized, standardized study conditions. Accordingly, real-world studies are indispensable because they show how unstable standardized performance results may be. Because there are no standardized conditions under real-world conditions, real-world data are very much needed in order to recognize the limits of applied diagnostic assays. Therefore, they provide an indispensable addition to standardized evaluation studies rather than just an acceptable weakness. Because low prevalence is the standard in the COVID-19 pandemic due to preventive countermeasures, it makes sense to accept a small number of positive samples in the interest of a realistic assessment. This is the only way to make realistic conclusions about the reliability of the test results.

## Figures and Tables

**Table 1:** Sensitivity, specificity and agreement between the two assessed antigen tests.

	PCR (*N* = 444)	NADAL (*N* = 215 *)	LumiraDx (*N* = 444)
Positives (%)	11 (2.5%)	8 (3.7%)	7 (1.6%)
Negatives (%)	433 (97.5%))	164 (76.3%)	437 (98.4%)
Sensitivity (0.95 CI)	1	0.1429 ** (0.0194, 0.5835)	0.4545 (0.2022, 0.7326)
Specificity (0.95 CI)	1	0.7644 ** (0.7016, 0.8174	0.9954 (0.9817, 0.9988)
Cohen’s kappa (0.95 CI) (*N* = 202)		0.060 (0.002, 0.167)	

* Forty-three samples were neither positive nor negative (borderline), ** estimation of sensitivity and specificity is based on all tested samples including borderline samples that were neither positive nor negative and therefore decreased diagnostic performance.

**Table 2:** Assessment of the matching of positive and negative tests with PCR Ct values.

PCR Target	Mean (SD), Median (Min, Max)				Mean (SD), Median (Min, Max)			
	NADAL				LumiraDx			
	*n*	Positive	*n*	Negative	*n*	Positive	*n*	Negative
T1 (Roche)	0		2	26.745 (8.14), 26.75 (20.99, 32.5)	4	22.44 (2.98), 21.04 (20.79, 26.91)	3	31.38 (1.1), 31.33 (30.31, 32.5)
T2 (Roche)	0		2	27.15 (8.56), 27.15 (21.09, 33.2)	4	21.89 (3.59), 21.05 (18.5, 26.97)	3	32.26 (1.3), 32.81 (30.78, 33.2)
ORF-1 (Genesig)	1	21.4 (-), 21.4 (21.4, 21.4)	2	35.18 (1.97), 35.18 (33.79, 36.57)	2	21.35 (0.07), 21.35 (21.3, 21.4)	4	33 (2.79), 32.56 (30.31, 36.57)
E-gene (Cepheid)	0		3	32.28 (2.58), 32.3 (29.7, 34.85)	0		5	32.09 (1.98), 32.3 (29.7, 34.85)
N2-target (Cepheid)	0		2	33.8 (1.84), 33.8 (32.5, 35.1)	0		2	33.8 (1.84), 33.8 (32.5, 35.1)

**Table 3:** Test characteristics as calculated by LCA and agreement of the TMA assay and the Genesig real-time PCR assay.

	TMA (*n* = 322)	Genesig Real Time PCR (*n* = 322)
Positives (%)	23 (7.1%)	21 (6.5%)
Negatives (%)	299 (92.9%)	301 (93.5%)
Sensitivity (0.95 CI)	0.7157 (0, 1)	0.6544 (0.4439, 0.8179)
Specificity (0.95 CI)	0.9997 (n.e.)	0.9999 (0, 1)
Cohen’s kappa (0.95 CI) (*n* = 322)	0.659 (0.492, 0.825)	

n.e. = not estimable. *n* = number. 0.95 CI = 95% confidence interval. TMA = transcription-mediated amplification.

**Table 4:** Ct values of the Genesig real-time PCRs for samples concordantly positive by TMA and real-time PCR as well as positive by real-time PCR only.

Parameter	Mean (SD), Median (Min, Max)			
	*n*	TMA Positive	*n*	TMA Negative
Genesig Ct value Mean (SD), Median (Min, Max)	15	30.808 (4.913), 31.8 (22.98, 37.91)	6	38.787 (0.944), 38.26 (38.07, 40.00)

SD = standard deviation. Min = minimum. Max = maximum. n = number. Ct = cycle threshold. TMA = transcription-mediated amplification.

**Table 5:** Sensitivity of TMA and Genesig real-time PCR of samples with low viral loads (Genesig Ct > 30) after 1:10 pooling.

	TMA (*n* = 18)	Genesig (*n* = 18)
Positives (%)	15 (83.33%)	6 (33.33%)
Negatives (%)	3 (16.67%)	12 (66.67%)
Sensitivity (0.95 CI)	0.8333 (0.568, 0.950)	0.3333 (0.148, 0.589)
Cohen’s kappa (0.95 CI) (*N* = 322)	0.182 (−0.036, 0.399)	

*n* = number. 0.95 CI = 95% confidence interval. TMA = transcription-mediated amplification.

**Table 6:** Ct values of the Genesig real-time PCRs still positive after pooling before and after the pooling step.

Parameter	Mean (SD), Median (Min, Max)			
	*n*	With 1:10 Pooling	*n*	Without Pooling
Genesig Ct value Mean (SD), Median (Min, Max)	6	38.223 (1.951), 38.555 (34.78, 40.00)	6	34.025 (1.980), 34.535 (31.44, 35.98)

SD = standard deviation. Min = minimum. Max = maximum. *n* = number. Ct = cycle threshold. TMA = transcription-mediated amplification.

**Table 7:** Predictive values as calculated for the assessed assays in the low prevalence setting in which they were evaluated.

Assay	Positive Predictive Value (0.95 CI)	Negative Predictive Value (0.95 CI)
NADAL	0.0966 (0.0131, 0.3943)	0.9768 (0.8965, 1)
LumiraDx	0.7102 (0.3159, 1)	0.9863 (0.9727, 0.9897)
TMA	1 (0, 1)	0.9690 (n.e.)
Genesig real-time PCR	1 (0.6488, 1)	0.9630 (0, 1)

## Data Availability

All relevant data are provided in the manuscript text.
